# Magnetic and Fluorescent Dual-Labeled Genetically Encoded Targeted Nanoparticles for Malignant Glioma Cell Tracking and Drug Delivery

**DOI:** 10.3390/pharmaceutics15102422

**Published:** 2023-10-04

**Authors:** Anna N. Gabashvili, Nelly S. Chmelyuk, Vera V. Oda, Maria K. Leonova, Viktoria A. Sarkisova, Polina A. Lazareva, Alevtina S. Semkina, Nikolai A. Belyakov, Timur R. Nizamov, Petr I. Nikitin

**Affiliations:** 1 Prokhorov General Physics Institute of the Russian Academy of Sciences, 38 Vavilov Street, 119991 Moscow, Russia; gabashvili_a@nsc.gpi.ru (A.N.G.);; 2 Laboratory “Biomedical Nanomaterials”, National University of Science and Technology “MISIS”, Leninskiy Prospekt 4, 119049 Moscow, Russia; 3 Department of Medical Nanobiotechnology, Pirogov Russian National Research Medical University, 1 Ostrovityanova Street, 117997 Moscow, Russia; lazareva_pa@rsmu.ru (P.A.L.);; 4 MILLAB Group Ltd., 100/2 Dmitrovskoe Highway, 127247 Moscow, Russia; 5 Department of Physical Chemistry, National University of Science and Technology “MISIS”, Leninskiy Prospekt 4, 119049 Moscow, Russia; 6 Biology Faculty, Lomonosov Moscow State University, 1 Leninskiy Gory, 119234 Moscow, Russia; 7 Cell Proliferation Laboratory, Engelhardt Institute of Molecular Biology RAS, 32 Vavilov Street, 119991 Moscow, Russia; 8 Department of Basic and Applied Neurobiology, Serbsky National Medical Research Center for Psychiatry and Narcology, 23 Kropotkinskiy Lane, 119991 Moscow, Russia

**Keywords:** encapsulins, fluorescence, iron biomineralization, target delivery system, magnetic particle quantification

## Abstract

Human glioblastoma multiforme (GBM) is a primary malignant brain tumor, a radically incurable disease characterized by rapid growth resistance to classical therapies, with a median patient survival of about 15 months. For decades, a plethora of approaches have been developed to make GBM therapy more precise and improve the diagnosis of this pathology. Targeted delivery mediated by the use of various molecules (monoclonal antibodies, ligands to overexpressed tumor receptors) is one of the promising methods to achieve this goal. Here we present a novel genetically encoded nanoscale dual-labeled system based on *Quasibacillus thermotolerans* (Qt) encapsulins exploiting biologically inspired designs with iron-containing nanoparticles as a cargo, conjugated with human fluorescent labeled transferrin (Tf) acting as a vector. It is known that the expression of transferrin receptors (TfR) in glioma cells is significantly higher compared to non-tumor cells, which enables the targeting of the resulting nanocarrier. The selectivity of binding of the obtained nanosystem to glioma cells was studied by qualitative and quantitative assessment of the accumulation of intracellular iron, as well as by magnetic particle quantification method and laser scanning confocal microscopy. Used approaches unambiguously demonstrated that transferrin-conjugated encapsulins were captured by glioma cells much more efficiently than by benign cells. The resulting bioinspired nanoplatform can be supplemented with a chemotherapeutic drug or genotherapeutic agent and used for targeted delivery of a therapeutic agent to malignant glioma cells. Additionally, the observed cell-assisted biosynthesis of magnetic nanoparticles could be an attractive way to achieve a narrow size distribution of particles for various applications.

## 1. Introduction

Human glioblastoma multiforme (GBM) is the most common primary brain tumor in adult patients, accounting for 45.2% of all malignant primary brain and central nervous system (CNS) tumors [1]. GBM can either arise de novo (in 80% of cases, occur in elderly patients with a mean age of 62 years) or develop from lower-grade astrocytomas or oligodendrogliomas (occur in younger patients with a mean age of about 45 years) [2,3,4].

GBM cells have many genetic and epigenetic mutations, which leads to a high rate of tumor cell proliferation, intensive neoangiogenesis, and invasion [5]. In addition, GBM is characterized by the multidrug resistance phenomenon, which occurs due to the expression of the ATP binding cassette of the family transporter proteins in cells [6,7]. As a result of ongoing chemotherapy, tumor cells undergo subcloning, with the subsequent emergence of a population of cells with increased chemoresistance [8]. When such a scenario is realized, the tumor recurs, and its growth and invasion become even more aggressive [9,10].

In light of the above, it is obvious that the development of new, more accurate approaches for the treatment of this pathology is an extremely urgent task. Among the emerging methods are, for example, CAR-T cell-based therapy [11], oncolytic virotherapy [12], antitumor vaccines [13], as well as the use of high-intensity focused ultrasound, a method that temporarily disrupts the blood-brain barrier (BBB) using pulsed ultrasound, which makes it possible to improve the efficiency of the delivery of therapeutic drugs to tumor cells [14].

A separate approach is the use of various nanosized vector systems for the targeted delivery of chemotherapeutic drugs to tumor cells [15,16,17]. In this case, various nanocarriers can be used, such as nanoparticles based on extracellular vesicles [18], lipids, polymers [19], gold [20,21], magnetic [22], viral, and drug-conjugated nanoparticles [23,24,25,26]. Of particular interest among those listed are protein-based bioinspired nanoparticles, such as viral nanoparticles, due to their biodegradability, as well as the possibility of functionalization of these structures by genetic transformation or chemical conjugation [27,28,29].

Encapsulins (or nanocompartments) are nanosized protein compartments exploiting biologically inspired designs capable of encapsulating dedicated protein cargos within the shells [30,31]. Nanocompartments are very homologous to viral capsids structurally, and they reliably protect the internal contents of the shell [32]. An interesting representative of encapsulins is the *Quasibacillus thermotolerans* (Qt) bacterium encapsulin due to its relatively large size (42 nm) and the ability to accumulate up to 23,000 iron atoms inside the shell. Qt encapsulin consists of a shell, self-assembled into an icosahedron from 240 identical protomer proteins and cargo IMEF (Iron-Mineralizing Encapsulin-Associated Firmicute) proteins with ferroxidase activity [33]. Under the action of IMEF, Fe^2+^ is oxidized to Fe^3+^ with the formation of iron-containing nanoparticles inside the shell [34]. Over the past few years, Qt encapsulin has found its application as a label for transmission electron microscopy (TEM) [34] and as a tool for cancer [35,36] and stem cells [37] monitoring via magnetic resonance imaging (MRI).

In this work, we present a new Qt encapsulin-based vector nanocontainer system genetically encoded in eukaryotic cells. First of all, we have developed a special cell line that can efficiently accumulate iron into Qt encapsulins due to the presence of the bivalent metal transporter mZip14. We used ferrous ammonium sulfate (FAS) as a source of Fe^2+^. We also modified the Qt encapsulin shell with an outward-facing Flag-tag that enabled purification of the Qt nanocompartments via immunoprecipitation. We next conjugated the resulting nanocontainer with human transferrin (Tf), preliminarily modified by fluorescein-6 (FAM) maleimide. Thus, we succeeded in obtaining a protein nanocarrier with a magnetic label inside the shell and a fluorescent label on the surface. The developed nanoplatform can be used to track glioma cells (as well as other tumor cells with a high level of transferrin receptors (TfR) expression) by MRI, magnetic particle quantification (MPQ) [38,39,40], or optical methods, and additionally supplemented with a therapeutic agent for targeted delivery to malignant glioma cells.

## 2. Materials and Methods

### 2.1. Cell Culture

All cell lines were grown under standard culture conditions (37 °C and 5% CO_2_). The human glioblastoma-astrocytoma cell line (U-87 MG, ATCC) was cultured with DMEM (Gibco, Waltham, MA, USA) medium supplemented with antibiotics (100 U/mL penicillin, 100 mg/mL streptomycin, Gibco), L-glutamine (2 mM, Gibco), and 10% FBS (fetal bovine serum, HyClone, Cytiva, Washington, DC, USA). HT1080 human fibrosarcoma cells (ATCC) were cultured in no phosphates DMEM (Gibco), supplemented with antibiotics (100 U/mL penicillin, 100 mg/mL streptomycin, Gibco), L-glutamine (2 mM, Gibco), and 10% FBS (Biowest, Riverside, MO, USA). Human adipose-derived mesenchymal stem cells (hMSCs) kindly provided by the Institute for Regenerative Medicine of Lomonosov Moscow State University (collection ID MSC_AD_MSU, www.human.depo.msu.ru, accessed on 17 August 2023). Cells were cultured in DMEM/F12 (Gibco) growth medium supplemented with 10% fetal bovine serum (HyClone), 2 mM L-glutamine (Gibco), and antibiotics (100 U/mL penicillin, 0.1 mg/mL streptomycin, Gibco).

### 2.2. Lentiviral Transduction of HT1080 Cells

Transduction of HT1080 fibrosarcoma cells with the QtEncFLAG-QtIMEF and mZip14 encoding lentiviral vectors [36] was performed according to standard protocol in DMEM growth medium supplemented with 10% heat-inactivated FBS and polybrene (8 µg/mL, Sigma-Aldrich, Darmstadt, Germany). Viruses were added to give a multiplicity of infection 4 for each vector. 48 h after transduction, the selection was started using puromycin (Thermo Fisher Scientific, Waltham, MA, USA) at a concentration of 3 µg/mL, and the medium with puromycin was changed to a new one every other day.

### 2.3. Reverse Transcription Polymerase Chain Reaction (RT-PCR)

HT1080-Qt cells were cultured to near confluence. The total RNA was then extracted by Extract RNA reagent (Evrogen, Moscow, Russia) according to the manufacturer’s instructions. RNA concentration was assessed by spectrophotometry. Then, cDNA was synthesized via SuperScript III Reverse Transcriptase (Thermo Fisher Scientific), oligo-DT, and random primers. The cDNA and negative control (RNA with no reverse transcriptase added) were used for classical PCR with Taq polymerase (Fermentas, Waltham, MA, USA). The products of PCR were separated in a 1% agarose gel electrophoresis. The amplified fragments were identified by their length.

### 2.4. Magnetic-Activated Cell Sorting (MACS)

The magnetic sorting of HT1080-Qt cells after 24 h of incubation with 1 mM FAS (Sigma-Aldrich) was performed using a magnetic separation kit (Miltenyi Biotec, Bergisch Gladbach, North Rhine-Westphalia, Germany) [41]. The cells were thoroughly washed with DPBS from FAS, detached from the plastic by trypsinization, centrifuged (500× *g*, 5 min), and resuspended in 1.5 mL of DPBS containing 2% FBS. The number of cells was counted using an automatic cell counter. MS column was placed into OctoMACS Separator and equilibrated with 0.5 mL PBS. The cell suspension was applied to a magnetic column, and free-passing cells were collected in a 15 mL tube. The column was washed twice with 1 mL of DPBS containing 2% FBS, removed from the separator’s magnetic field, and transferred in a new 15 mL tube. Next, 1 mL of DPBS with 2% FBS was added to the column, and the cells retained in the column were eluted using a plunger. Then, the cells were centrifuged (500× *g*, 5 min), resuspended in a complete DMEM, and counted. Control HT1080 cells were processed according to the same protocol.

### 2.5. Cytotoxicity Assay

The effect of FAS on HT1080 and HT1080-Qt cell viability was determined using the CellTiter 96 MTS (3-(4,5-carboxyme-thoxyphenyl)-2-(4-sulfophenyl)-2H-tetrazolium) cell proliferation assay kit (Promega, Madison, WI, USA). The assay was carried out according to the manufacturer’s instructions [42]. The cells were cultured in 96-well plates (~10.000 cells/well, Corning, New York, NY, USA) in 100 µL of DMEM medium supplemented with 10% FBS 24 h prior to the assay. Then, FAS was added to the cells at a concentration range of 0.6–10 mM. Cells incubated with the culture medium were used as a positive control. After 24 h of incubation, a fresh growth medium with MTS reagent was added to each well. The cells were incubated with MTS reagent for 4 h in standard conditions. Optical density (OD) was measured using a Multiscan GO plate reader (Thermo Fisher Scientific), λ = 490 nm. Cell viability (%) was calculated as:

Cell viability = (As − Ab/Ac − Ab) × 100%

where As—mean OD in sample wells, Ab—mean OD in blank wells, and Ac—mean OD in positive control wells.

To assess the potential cytotoxic effect of Qt and Qt-Tf-FAM on MSCs and U87-MG cells, resazurin (Sigma-Aldrich, Darmstadt, Germany) assay was provided according to the manufacturer’s instructions (Supplementary Figure S3). Cells were seeded on opaque-walled 96-well plates at ~10.000 cells/well in full DMEM medium. Qt and Qt-Tf-FAM conjugates were added to the cells at a concentration range of 31.2–500 ng/µL for 24 h. After 24 h of incubation, a fresh growth medium (100 μL/well) with 44 μM resazurin sodium solution was added to each well. After 4 h of incubation, the fluorescence of resorufin was recorded using a CLARIOstar Plus plate analyzer (BMG LABTECH, Ortenberg, Germany), λ = 560 nm. The number of metabolically active cells was calculated using the following formula:

Number of metabolically active cells = (As − Ab/Ac − Ab) × 100%

where As is the average fluorescence intensity value in sample wells, Ab—average fluorescence intensity value in blank wells (no cells added), and Ac—average fluorescence intensity value in control wells (untreated cells).

### 2.6. Transmission Electron Microscopy (TEM)

HT-1080-Qt cells (4 × 10^4^ cells/well) were seeded into 8-well µ-Slide (Ibidi, Grafelfing, Germany) and cultured in full DMEM-F12 medium supplemented with 1 mM FAS for 24 h. Next, the cells were thoroughly washed with DPBS and fixed with 2% paraformaldehyde and 2.5% glutaraldehyde (Sigma-Aldrich) solution in PBS, pH 7.4. Then, the cells were postfixed with 1% osmium tetroxide and dehydrated in ethanol of increasing concentrations (50%, 70%, 80%, 95%). At last, the cell sample was embedded in an Epoxy resin using an Epoxy embedding medium kit (Sigma Aldrich) according to the manufacturer’s instructions. Ultrathin cell sections were obtained with an EM UC6 ultramicrotome (Leica, Wetzlar, Germany). 

A suspension of purified Qt encapsulins in TBS (tris-buffered saline) was dropped onto the surface of a formvar-coated copper grid (300 mesh), and the solvent was evaporated. TEM analysis was performed on a JEOL JEM-1400 (Akishima, Tokyo, Japan) microscope. The average size of iron-containing nanoparticles in Qt shells was calculated from images by analysis of 100 nanoparticles using ImageJ 1.53v software, with nonlinear approximation fitting using log-normal distribution in GraphPad Prism 6.01.

### 2.7. Immunoprecipitation

HT1080-Qt cells were cultured in 6-well plates in no phosphate DMEM culture medium supplemented with 10% FBS to near confluence. Then, FAS (1 mM) was added to the cells for 24 h. Next, the cells were washed with DPBS and lysed with MPER (mammalian protein extraction reagent) cell lysis buffer (50 mM Tris HCl, 150 mM NaCl, 1% Triton X-100) for 15–20 min at 4 °C on a shaker. Cell lysates were centrifugated (10,000× *g*, 30 min, at 4 °C), and the supernatant was incubated with pre-equilibrated Anti-DYKDDDDK Tag (L5) Affinity Gel (BioLegend, San Diego, CA, USA) for 2 h on a shaker at 4 °C. For elution, the gel was incubated with 100 μg/mL 3× FLAG Peptide (Sigma-Aldrich) for 30 min at 4 °C and centrifugated at 5000× *g* for 2 min. The eluate was kept at 4 °C in TBS.

### 2.8. Tf-FAM Labeled Qt Encapsulins Engineering

To obtain Tf-FAM conjugate, 10 mg of human apotransferrin (Merck, Rahway, NJ, USA, MW ~80 kDa) was dissolved in 2 mL of DPBS (pH = 6.8), 2 µL of fluorescein-6 (FAM) maleimide (Lumiprobe, Russia) (20 mg/mL in DMSO) was added to transferrin and mixed at constant stirring for 16 h, RT (room temperature). The resulting solution was purified 5 times using 30 kDa MWCO (molecular weight cut off) Amicon filters (Millipore Billerica, MA, USA) and adjusted to 1 mL in PBS (pH = 7.4). Next, 10 μL of 6-Maleimidohexanoic acid N-hydroxysuccinimide ester (0.5 mg/mL, water: DMSO 1:1, Merk) was added to Tf-FAM and incubated for 1 h, RT. The solution was then purified 5 times by 30 kDa MWCO Amicon filters and resolved in 1 mL PBS (pH = 6.8). At the last stage, isolated Qt encapsulins were resolved in PBS (pH = 6.8) and mixed in a ratio of 1:1 by weight in 1 mL of the reaction mixture for 16 h. The resulting conjugates were purified 7 times via 100 kDa MWCO Amicon filters, 500 ng/μL stock solution of Qt-Tf-FAM conjugates stored at 4 °C until further experiments.

### 2.9. Dynamic Light Scattering

ZetaSizer Nano ZS (Malvern, UK) was used to determine the hydrodynamic size of isolated Qt encapsulins. Measurements were performed at 25 °C using a rectangular glass cuvette containing 1000 μL of protein solution (eluate was diluted in TBS 1:4 rate). Volume nanoparticle size distribution data was automatically plotted using Malvern Zetasizer Software (v. 3.30). 

### 2.10. Prussian Blue Staining

Prussian blue staining of HT1080-Qt cells after 24 h incubation with 1 mM FAS, or hMSCs and U87 cells after 90 min incubation with iron-loaded Qt/Qt-Tf-FAM encapsulins (30 µL PBS containing Qt-Tf-FAM and Qt pre-aligned for protein concentration (~500 ng/µL) was added to 2 × 10^4^ cells) was carried out using Iron Stain Kit (Sigma-Aldrich), according to the manufacturer’s instructions. Images of the cells were taken with an inverted microscope Primo Vert (Zeiss, Oberkochen, Germany).

### 2.11. Measurement of Intracellular Iron Content by Atomic Emission Spectroscopy (AES)

hMSCs and U87 cells were seeded in 6-well culture plates (Corning, 3 × 10^5^ cells/well) and cultured for 24 h. Next, 100 μL/well of PBS containing Qt or Qt-Tf-FAM encapsulins pre-aligned by protein concentration (~500 ng/μL) was added to the cells in 1200 μL of growth medium to the final concentration of 42 ng/μL and incubated for 90 min. After that, the cells were thoroughly washed with DPBS, detached from the plastic, precipitated by centrifugation, and counted. 5.6 × 10^5^ cells were dissolved in 50 µL of concentrated nitric acid for 2 h at 60 °C. Iron concentration was determined using a 4200 MP-AES atomic emission spectrometer (Agilent, Santa Clara, CA, USA). The experiment was carried out in three repetitions.

### 2.12. Laser Scanning Confocal Microscopy

U87 cells were seeded on 3.5 cm µ-Dishes with a polymer coverslip bottom at 1 × 10^4^ cells/dish for confocal microscopy and cultured for 48 h in standard conditions. Following the cultivation, 40 μL of Qt-Tf-FAM stock solution was added to the cells in 70 μL of cell culture media to the final concentration of 286 ng/μL. The cells were incubated at 37 °C and 5% CO_2_ for 30 min, 60 min and 120 min. Additionally, LysoTracker Deep Red (Ex-Max 647 nm/Em-Max 668 nm) dye (Thermo Fisher Scientific) was added to the cells to a final concentration of 50 nM for 30 min. Then, the medium was removed, and the cells were rinsed twice with DPBS (Gibco). The cells were imaged using a Zeiss laser scanning confocal microscope equipped with LSM 710 NLO scanning system, 40× water immersion objective lens, and 458, 488, 514, 543, 561, 594, and 633 nm lasers. Scanning was performed using the Carl Zeiss LSM-710-NLO ZEN 2010 software.

### 2.13. Magnetic Particle Quantification 

For the detection of superparamagnetic or ferromagnetic fraction of iron oxide nanoparticles in the samples, the magnetic particle quantification (MPQ) method was used. The MPQ employs a nonlinear magnetization of such particles subjected to a magnetic field at frequencies *f*_1_ = 100 Hz and *f*_2_ = 100 kHz, recording the magnetic particle (MP) response at the combinatorial frequency *f*_2_ *+* 2*f*_1_ [39]. The method is insensitive to dia- and paramagnetic materials that have a linear dependence of the magnetization on the external magnetic field, such as endogenous forms of iron, etc. The related readers offer the record limit of detection of 0.4 ng of MP in 0.2 mL volume or 39 pg of vortex magnetic nanostructures in an extremely wide 7-order linear dynamic range and demonstrate high sensitivity for in vitro assays [43,44], studies of the circulating kinetics of various MPs in the bloodstream [16,45], monitoring of MP biodegradation in vivo [46,47].

## 3. Results

### 3.1. Iron Biomineralization Inside the Encapsulins

**Figure 1:**
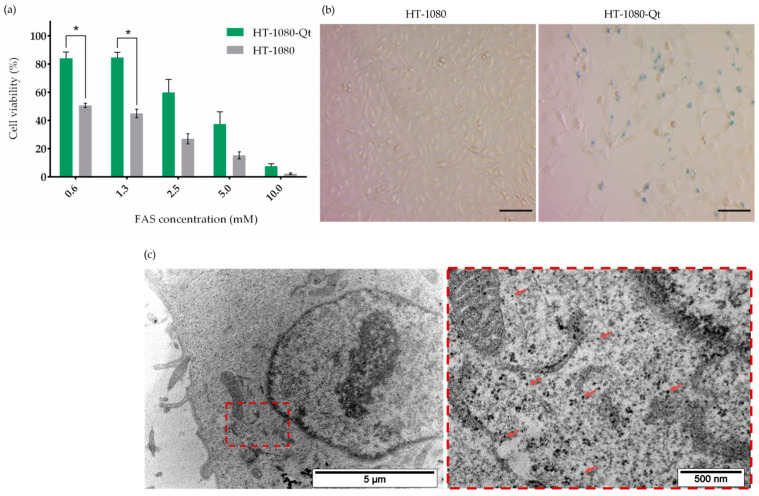
Sequestration of iron within Qt encapsulins in HT-1080-Qt cells. (**a**) MTS assay evaluating the cytotoxicity of various FAS concentrations in HT-1080-Qt and HT-1080 cells. The data are shown as the mean ± S.D. of three independent experiments; *p*-values were calculated using a one-tailed *t*-test, assuming unequal variances (* indicate *p*-values < 0.05). (**b**) Prussian blue staining of HT-1080 and TH-1080-Qt cells after incubation with 1 mM FAS for 24 h. White-field microscopy, Zeiss Primo Vert, scale bars are 50 μm. (**c**) Bright-field TEM image of an ultrathin section of HT-1080-Qt cells after 24 h of incubation with 1 mM FAS; red arrows indicate iron oxide cores inside encapsulins shells. Scale bars are 5 μm and 500 nm (close-up of an exemplary region).

Taking into account that the formation of iron-containing nanoparticles in HT1080-Qt cells requires preliminary incubation with FAS for 24 h, we carried out an assessment of the viability of HT1080-Qt cells in the presence of FAS at various concentrations (from 0.6 to 10 mM), where HT1080 cells were used as a control. From the histogram presented in Figure 1a, it is clearly seen that HT1080 cells are sensitive to the extra iron in the growth medium, and in the presence of FAS, a dose-dependent decrease in cell viability is observed. However, the viability of HT1080-Qt cells at the same concentrations of FAS is higher—statistically significant differences (*p* < 0.05) are observed at concentrations of 0.6 mM and 1.3 mM. Thus, based on the viability assay results, we chose a FAS concentration of 1 mM for all further studies. Next, Prussian blue staining was performed in order to qualitatively assess the accumulation of iron in HT1080-Qt cells after incubation with FAS (Figure 1b). The resulting micrographs of HT1080-Qt cells show a characteristic blue color with cytoplasmic localization, indicating the accumulation of iron deposits. In control HT1080 cells, no coloration was observed. The formation of iron-containing nanoparticles in Qt encapsulins was also detected by TEM (Figure 1c). The micrograph clearly shows multiple electron-dense nanostructures diffusely located in the cytoplasm of the cell.

### 3.2. Encapsulins Assisted Production of Iron Oxide Nanoparticles with Nonlinear Magnetization

**Figure 2:**
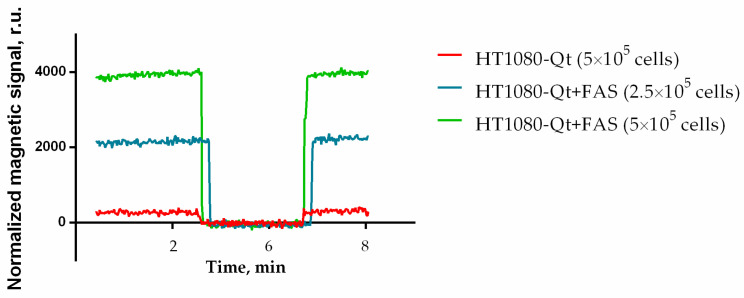
The magnetic signals of the control (HT1080-Qt cells) and two samples with different concentrations of cells preincubated with FAS measured using the MPQ method. Initial and final signal levels: samples are inside the coils; intermediate level—samples are outside the MPQ coils.

Experiments have shown that iron biomineralization within encapsulins, as described in session 3.1 above, allows the production of nanoparticles that are highly detectable by the MPQ technique. This indicates a nonlinear dependence of their magnetization on the external magnetic field, which is characteristic of the superparamagnetic or ferromagnetic behavior of iron oxide nanoparticles. Figure 2 demonstrates the magnetic signals of the control (HT1080-Qt cells) and two samples with different concentrations of cells preincubated with FAS (2.5 × 10^5^ and 5 × 10^5^ HT1080-Qt cells). It is clearly shown that the signals of HT1080-Qt + FAS with 5 × 10^5^ cells are 12 times larger than the signals of the control sample.

Such cell-assisted biosynthesis of well-calibrated magnetic nanoparticles is of great fundamental and practical interest if high productivity can be achieved by proper process optimization. In some sense, this is an interesting process, the opposite of that studied in Ref. [47], where, on the contrary, it was shown that nonlinear superparamagnetic nanoparticle degradation in vivo significantly increases the expression of iron-related proteins such as ferritin, ferroportin, divalent metal transporter 1 (DMT1), hemoglobin, etc., which are paramagnetic and have a linear dependence of the magnetization on the external magnetic field.

### 3.3. Qt Encapsulins Isolation, Characterization and Modification

**Figure 3:**
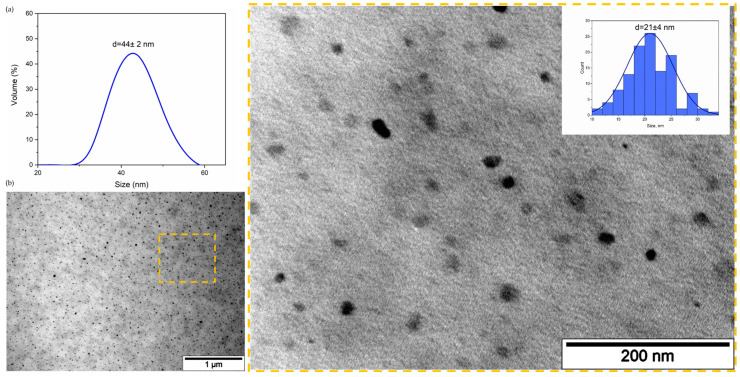
Qt encapsulins isolation and characterization. (**a**) Dynamic light scattering analysis of purified Qt encapsulins (44 ± 2 nm; PDI 0.2). Hydrodynamic size of Qt encapsulins, volume distribution. (**b**) Bright-field TEM image of iron-loaded isolated Qt encapsulins, scale bars are 1 μm and 200 nm (close-up region). The inset demonstrates the size distribution of iron cores inside the encapsulin shells.

Next, Qt encapsulins were purified from HT1080-Qt cells by the immunoprecipitation method using anti-Flag affinity gel for isolation. The hydrodynamic size of Qt encapsulins was determined by dynamic light scattering (DLS). Figure 3a shows an example of the size distribution of Qt encapsulins, where the average diameter of isolated Qt nanocompartments was 44 ± 2 nm. The isolated encapsulins were also visualized by TEM (Figure 3b), and the average size of iron-containing nanoparticles formed inside the shells was measured, and a narrow size distribution was observed (21 ± 4 nm). The hydrodynamic size of the isolated encapsulins, as well as the average size of iron-containing nanoparticles formed inside the shells of encapsulins, established in our study, are in good agreement with the literature data [34].

**Figure 4:**
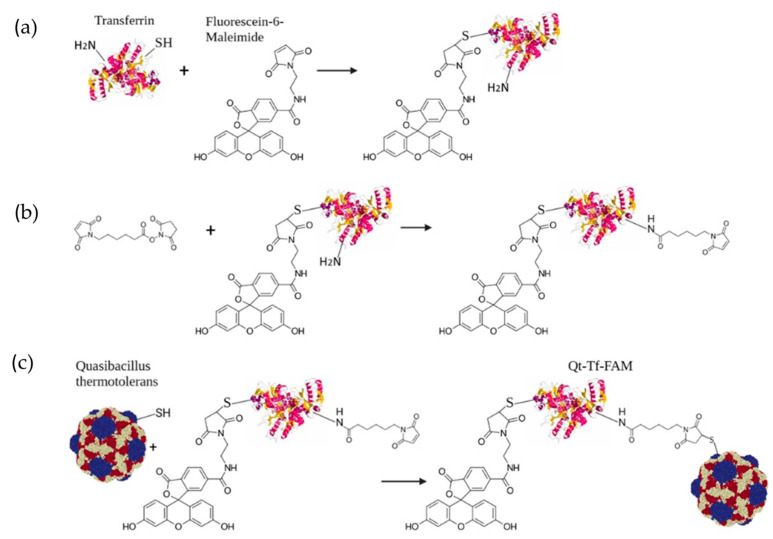
Reaction schemes for the preparation of Tf-FAM-labeled Qt encapsulins. (**a**) Transferrin conjugation with FAM; (**b**) Tf-FAM reaction with the EMCS linker; (**c**) Tf-FAM conjugation with Qt encapsulin shells via EMCS linker.

Isolated encapsulins were then chemically modified by conjugation with human Tf prelabeled with FAM (Figure 4).

Wherein the FAM label will allow us to obtain an additional fluorescent signal and to dynamically assess the internalization of Qt-Tf-FAM conjugates in U87 glioma cells using confocal microscopy.

### 3.4. Cellular Uptake of Dual-Labeled Vectorized Encapsulins by Glioma Cells

**Figure 5:**
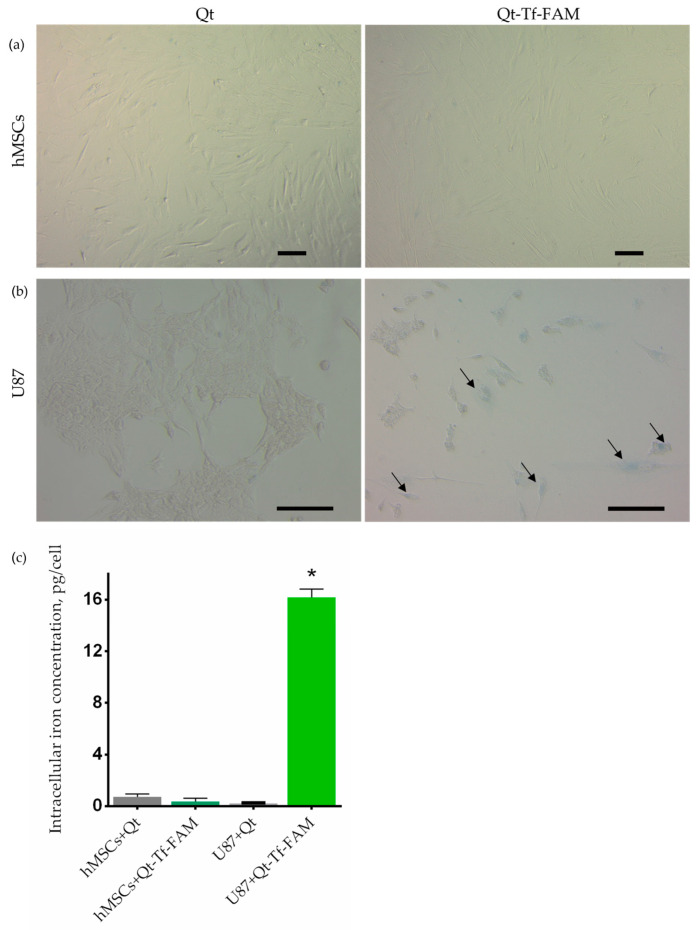
Cellular uptake of vectorized Qt encapsulins by hMSCs and U87 cells. Prussian blue staining of hMSCs (**a**) and U87 cells (**b**) after incubation with Qt or Qt-Tf-FAM for 90 min. Black arrows indicate the characteristic blue coloration of iron deposits in the cytoplasm and nucleus. White-field microscopy, Zeiss Primo Vert, scale bars are 50 μm and 100 μm, respectively. (**c**) Intracellular iron content in hMSCs and U87 cells was quantified by AES after incubation with Qt or Qt-Tf-FAM for 90 min. The data are shown as the mean + S.D of three independent experiments (* indicates *p*-value < 0.05).

At last, a comparative analysis of the uptake of iron-containing Qt and Qt-Tf-FAM encapsulins by U87 cells and hMSCs was carried out by qualitative and quantitative assessment of iron accumulation via Prussian blue staining and AES, respectively. As can be seen from the micrographs presented in Figure 5a, no coloration was registered after the incubation of hMSCs with Qt and Qt-Tf-FAM. In contrast, after incubation of U87 cells with Qt-Tf-FAM (Figure 5b), blue inclusions in the cell cytoplasm, indicating the accumulation of iron deposits, are clearly visible. These data are in good agreement with the quantitative results of assessing the accumulation of intracellular iron (Figure 5c) obtained by the AES method (0.2 ± 0.04 pg/cell and 16 ± 0.7 pg/cell after U87 cells incubation with Qt and Qt-Tf-FAM, respectively).

**Figure 6:**
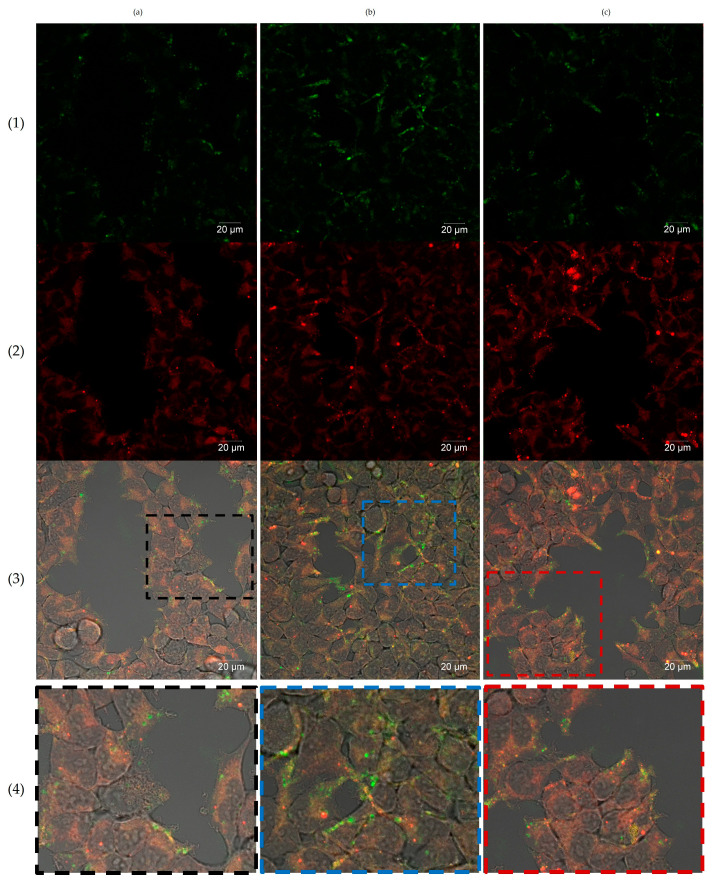
Confocal imaging of U87 glioma cells after (**a**) 30 min, (**b**) 90 min, (**c**) 120 min of incubation with Qt-Tf-FAM. (1) FAM (green fluorescent signal, Ex-Max 498 nm/Em-Max 517 nm), (2) LysoTracker Deep Red dye (red fluorescent signal, Ex-Max 647 nm/Em-Max 668 nm), (3) Merge, (4) Region of interest. Laser scanning confocal microscopy, Zeiss LSM 710 NLO, scale bars are 20 µm.

The internalization of Qt-Tf-FAM conjugates in U87 glioma cells was also analyzed by laser scanning confocal microscopy. It was shown that after the addition of the conjugates to the cells, a gradual accumulation of the FAM fluorescent signal in the cells occurs. After 30 min of incubation (Figure 6a), the fluorescent signal was localized on the cell membranes, followed by further time-dependent internalization of the conjugates into the cells. After 60 min of incubation (Figure 6b), the FAM fluorescent signal was clearly detected in the cell cytoplasm. Finally, after 120 min of incubation (Figure 6c), it could be seen that the part of the FAM signal was colocalized with the LysoTracker far-red fluorescence signal, which may indicate that the conjugate was delivered to the lysosomes.

Thus, the difference in the efficiency of cellular uptake of the obtained vector system by glioma cells and non-tumor cells was assessed, on the one hand, by qualitative and quantitative determination of the iron content in cells and, on the other hand, by confocal microscopy. Both approaches indicate the selectivity of binding of Qt-Tf-FAM conjugates to U87 glioma cells.

## 4. Discussion

An important task in the development of xenogenic proteins-based nanoplatforms is the assessment of the safety of the resulting product and, in particular, the assessment of immunogenicity. Currently, there are several works concerning the use of encapsulins in vivo. For example, in one of our studies [36], we demonstrated that implantation of murine 4T1 breast carcinoma cells stably expressing Qt encapsulin genes (4T1-Qt cells) led to the formation of tumors in mice, and the growth rate of tumors did not differ from that of tumors formed after injection of intact 4T1 cells. Rejection of 4T1-Qt cells was not detected in any of the mice in the experiment. Also, in a recent study [48], it was shown that the insertion of encapsulin encoding genes by injection of adeno-associated virus (AAV) into the mouse brain led to the expression of encapsulin in hippocampal neurons, which was confirmed by immunohistochemical fluorescence analysis. The authors of the work did not note any immune response from microglia. These data allow us to hope that the application of encapsulins in vivo can be safe. However, this issue, of course, remains open for discussion today. This problem can be partially overcome by PEGylation of nanocompartments. There are also several encapsulin-based targeted delivery systems. For example, one system selectively binds to human hepatocellular carcinoma cells [49] and the other [50] to breast cancer cells. However, the safety assessment of the in vivo use of the systems described above has not been carried out yet.

For the present study, a comparative analysis of iron accumulation in various transgenic cell lines containing Qt encapsulin genes was preliminarily carried out (some of the data obtained are presented in Supplementary Figure S2 and in [36]), and the HT1080-Qt cell line proved to be the most promising candidate out of six candidate lines obtained by us. We next hypothesized that the resulting HT1080-Qt cells are heterogeneous in the context of encapsulin synthesis, and the cells that synthesize encapsulins most efficiently would accumulate more iron and thus be retained in the magnetic column. Thereby, in this work, we used MACS to obtain a fraction of encapsulin-enriched HT1080-Qt cells.

A comparative viability assessment showed that in the presence of extra iron in the growth medium, encapsulins in HT1080-Qt cells play a protective role by sequestering iron in the cytoplasm, thereby reducing the amount of free cytoplasmic iron, which increases cell survival. Remarkable, a similar protective mechanism has also been described in encapsulin-expressing bacteria [51]. 

Promising results indicating that Qt-Tf-FAM selectively binds to U87 cells were also obtained by the atomic emission spectroscopy method, which agrees well with the laser scanning confocal microscopy data.

## 5. Conclusions

In summary, in this work, we obtained a new Qt encapsulin-based targeted delivery system that selectively binds to U87 glioma cells. The targeting of the developed system was studied by qualitative and quantitative assessment of intracellular iron accumulation, as well as by fluorescent microscopy. Both approaches confirm the selectivity of binding of the Qt-Tf-FAM system to U87 glioma cells. The nanocarrier can be further supplemented with a therapeutic drug, such as, for example, temodal, and used in studies on an in vivo model of glioma. In addition, the observed cell-assisted biosynthesis of superparamagnetic nanoparticles, which have a nonlinear dependence of magnetization on the external magnetic field and a very narrow size distribution, is of great interest. Optimization of this process can lead to high throughput production of such nanoparticles for fundamental research and various practical applications.

## Data Availability

Not applicable.

## Supplementary Materials

The following supporting information can be downloaded at https://www.mdpi.com/article/10.3390/pharmaceutics15102422/s1, Figure S1: Reverse transcription-polymerase chain reaction analysis of HT1080-Qt cells. Figure S2: Iron biomineralization in Qt encapsulin genes containing cell lines. Figure S3: Resazurin assay evaluating the cytotoxicity of Qt and Qt-Tf-FAM in various concentrations in U87 cells and hMSCs.
