# Supplementary material for: Magnetic and Fluorescent Dual-Labeled Genetically Encoded Targeted Nanoparticles for Malignant Glioma Cell Tracking and Drug Delivery
Source: Pharmaceutics. 2023 Oct 4;15(10):2422. doi: 10.3390/pharmaceutics15102422 (PMC10609955; doi:10.3390/pharmaceutics15102422)
Supplement: Supplementary file 1 [file pharmaceutics-15-02422-s001.zip › pharmaceutics-2592710-supplementary.pdf]

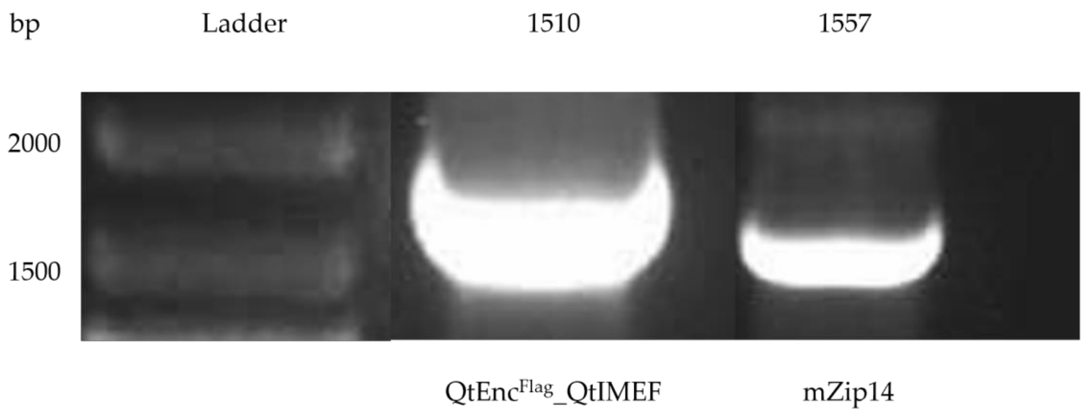

Figure S1. Reverse transcription-polymerase chain reaction analysis of HT1080-Qt cells.

Pharmaceutics **2023**, *15*, 2422. https://doi.org/10.3390/pharmaceutics15102422 www.mdpi.com/journal/pharmaceutics

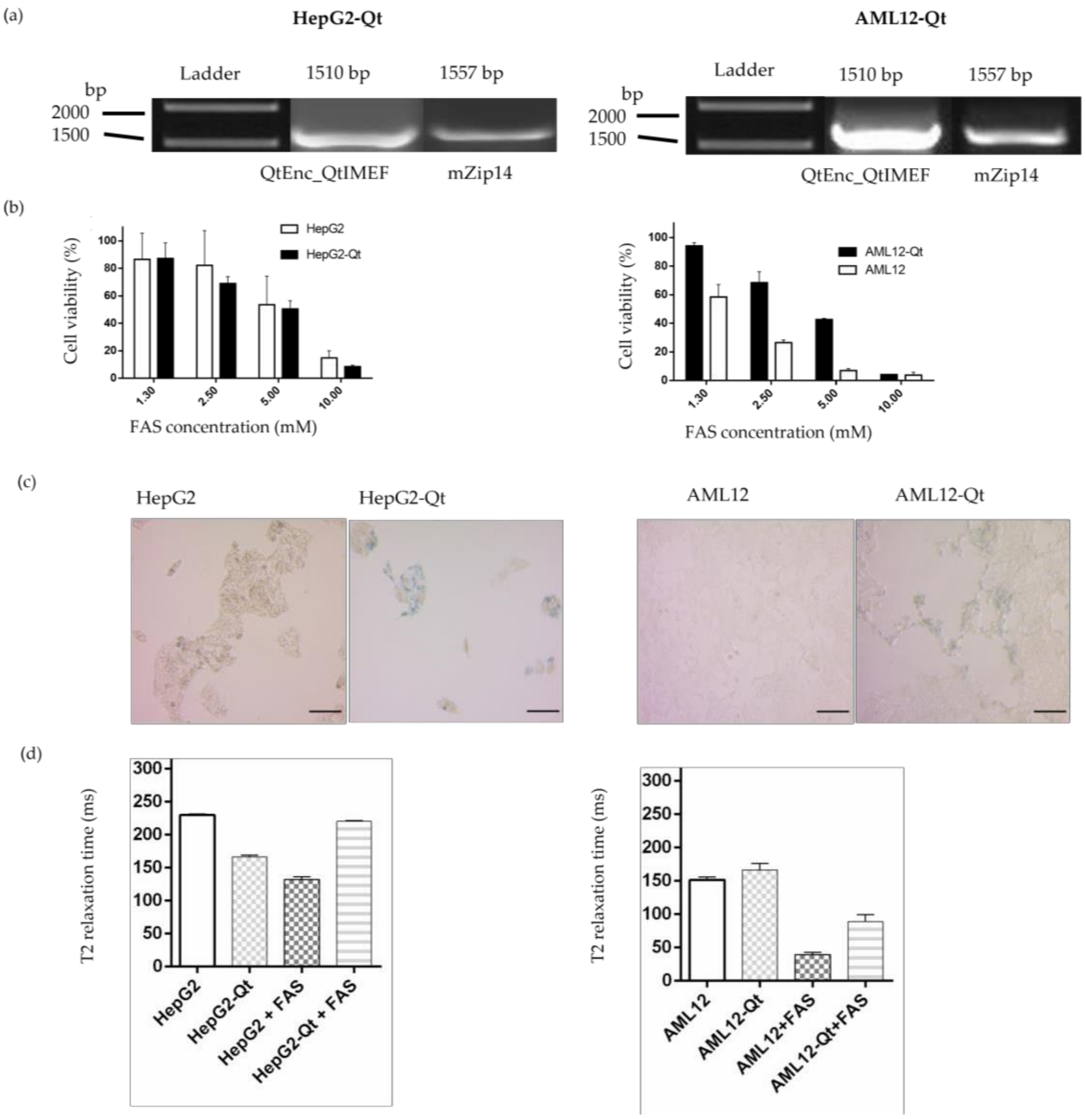

Figure S2. Iron biomineralization in Qt encapsulin genes containing cell lines: RT-PCR (a), viability assay (b), Prussian blue staining of cells after incubation with 2 mM FAS for 24 h (c) and MRI (d) analysis of HepG2-Qt and AML12-Qt cell lines.

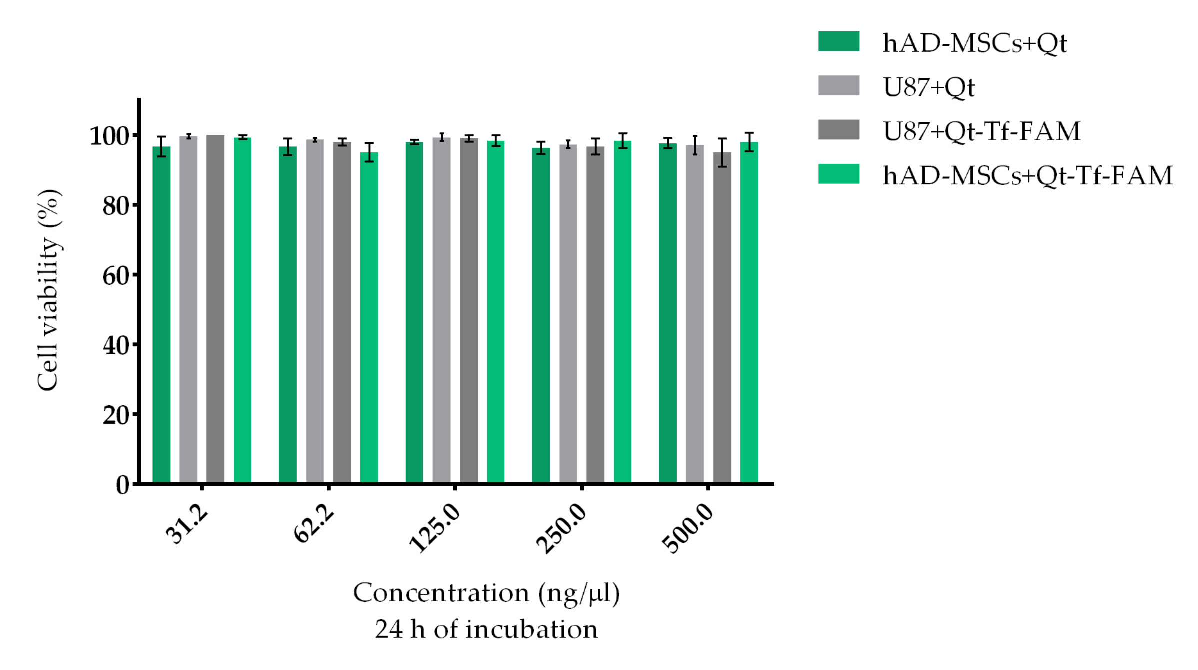

Figure S3. Resazurin assay evaluating the cytotoxicity of Qt and Qt-Tf-FAM in various concentrations in U87 cells and hMSCs.
